# Improving Accuracy of Real-Time Positioning and Path Tracking by Using an Error Compensation Algorithm against Walking Modes

**DOI:** 10.3390/s23125417

**Published:** 2023-06-07

**Authors:** Jiale Gong, Ziyang Li, Mingzhu Chen, Hong Wang, Dongmo Hu

**Affiliations:** 1 Department of Mechanical Engineering and Automation, Northeastern University, Shenyang 110819, China; 1610073@stu.neu.edu.cn (J.G.); 2110108@stu.neu.edu.cn (Z.L.); 2 Senzhigaoke Co., Ltd., Shenyang 110002, China; annehdm0204@163.com; 3 Robotics Laboratory, Shenyang Sport University, Shenyang 110102, China; mingzhu_chen@foxmail.com

**Keywords:** positioning, error compensation, plantar pressure, motion capture, WSN

## Abstract

Wide-range application scenarios, such as industrial, medical, rescue, etc., are in various demand for human spatial positioning technology. However, the existing MEMS-based sensor positioning methods have many problems, such as large accuracy errors, poor real-time performance and a single scene. We focused on improving the accuracy of IMU-based both feet localization and path tracing, and analyzed three traditional methods. In this paper, a planar spatial human positioning method based on high-resolution pressure insoles and IMU sensors was improved, and a real-time position compensation method for walking modes was proposed. To validate the improved method, we added two high-resolution pressure insoles to our self-developed motion capture system with a wireless sensor network (WSN) system consisting of 12 IMUs. By multi-sensor data fusion, we implemented dynamic recognition and automatic matching of compensation values for five walking modes, with real-time spatial-position calculation of the touchdown foot, enhancing the 3D accuracy of its practical positioning. Finally, we compared the proposed algorithm with three old methods by statistical analysis of multiple sets of experimental data. The experimental results show that this method has higher positioning accuracy in real-time indoor positioning and path-tracking tasks. The methodology can have more extensive and effective applications in the future.

## 1. Introduction

High-precision 3D indoor positioning and navigation methods [1] have a strong demand for portability, real-time, high accuracy and stability in scenarios such as the safety and security of rescue personnel and real-time worker location identification in industrial scenes. However, with the improvement of social modernization and the complexity of urban building structures, traditional navigation modes based on radio frequency navigation are difficult to meet the existing needs. Radio frequency navigation methods rely on satellite timing positioning or signal reduction algorithms for spatial positioning [2,3,4]. Traditional civil frequency satellite positioning methods have poor positioning accuracy, with an accuracy range of about 2 m. Although RTK or PPK technology based on differential satellite positioning [5,6,7] has been applied in recent years, and its accuracy has also been improved to a centimeter level. For rescue and industrial indoor scenes, satellite signals cannot be effectively transmitted and received [8], which greatly limits the application and development of this technology in indoor environments. However, various indoor navigation technologies based on signal attenuation models, such as positioning based on WIFI and UWB ultra-bandwidth [9,10], suffer from huge installation costs and relatively complex usage mode. The use of this type of approach does not allow for high indoor positioning accuracy and requires dense pre-set dedicated RF tag nodes, with positioning accuracy being greatly affected by node density and line-of-sight obstructions.

In the context of the rapid development of MEMS technology [11], especially with respect to human wearable devices [12], the excellent characteristics of MEMS, such as miniaturization and low power consumption, make it widely demanded and applied in medical, industrial modernization, military, sport and other scenarios. Optical motion capture methods or MEMS sensor motion capture method is commonly used in precise positioning of the human body [13,14,15]. However, this positioning method based on special equipment for remote video acquisition cannot be effectively applied in situations such as fire-damaged scenes or earthquake rescue in which the space is small and the camera is not arranged in advance and cannot be possible to get enough good line of sight [16,17]. The inertial navigation positioning method can effectively avoid the limitation of relying on external pre-arranged equipment. This method relies on the built-in sensors of the inertial navigation system to sense the movement and rotation of the object and to obtain the moving position and trajectory of the three-dimensional space by algorithm [18] integration. However, the high-precision inertial navigation positioning method requires the use of a high-precision fiber optic [19] gyroscope or a high-precision accelerometer [20], which is too large for the human body to wear. Furthermore, the resolution circuit of the high-resolution accelerometer [21] is relatively complex, so it cannot be used for portable applications in body-worn devices.

The integrated inertial navigation method using MEMS manufacturing process [22] has the characteristics of small volume, light weight and simple structure. As a portable sensor scheme that can be worn on the human body, it is widely used in rescue scenarios and other applications, for example, a watch accessory or integrated into clothing [23]. As the precision of the monolithic integration sensor cannot be compared to that of larger light-based gyroscopes and piezoelectric accelerometers, the data collected by the MEMS sensor itself alone have a large drift in real-time position obtained by primary and secondary integration of angular velocity and acceleration due to manufacturing accuracy errors and low sampling frequency [24,25]. The traditional positioning method based on the monolithic MEMS sensor often use the zero velocity detection methods (ZUPT) [26], which relies on fixing the position of human lower limb leg or foot, by detecting the state of zero relative velocity between the foot and ground, such as the supporting phase of human gait to reduce the serious position deviation caused by sensor data drift. This method requires to analyze and preprocess the whole data to obtain the characteristics of the overall drift of the data, followed by data filtering to finally obtain more accurate positioning data. Nevertheless, as a replay-based positioning method, it does not allow for good real-time human location. Another solution is to use the method of whole-body wearable motion capture [27], which detects the location of the person wearing the device. This method can realize real-time positioning, but it can only rely on inertial data for landing detection on the foot. In the implementation of landing detection, the sensor fixed near the foot or lower limb may appear wrong landing detection, advance detection or lag landing detection due to occasional small sliding movements [28,29]. Therefore, how to design a positioning method suitable for the application scene with high accuracy, stability and real-time requirements is a problem that needs to solve for indoor positioning technology.

This paper focused on improving the positioning accuracy of gait analysis based on IMUs. By the motion capture system with 12 wireless IMU modules we implemented before, the real-time position of each foot can be got within 20 ms. We attempted to get more accurate position for real-time walking simulation, and it gave a try to add two high-resolution pressure insoles to the system, which could be used in a particular example of analyzing a footballer’s nifty footwork. The main aims and contributions of this paper are:

- Using high-spatial-resolution plantar-pressure insoles to capture plantar pressure distribution has improved the accuracy of plantar pressure distribution when the foot comes in contact with the ground, achieving recognition of five walking modes;
- Analyzing and comparing three previous IMU landing point capture and path tracking methods, conducting error analysis and proposing improved methods;
- Implementing a system with a fully wireless, synchronous and real-time transmission software and hardware architecture, and completing on-site testing of five walking modes;
- Exploring the possibility of multi-scene adaptation and proposing a method for determining the position of feet under changing ground conditions.

## 2. Related Work

The positioning method of relying on IMU can achieve spatial positioning. How to capture the time and position of touching the ground more accurately is the key to improve the accuracy of the spatial positioning of inertial motion capture. Common methods for judging the state of the foot touchdown are the feet ground height comparison method, foot acceleration jitter judgment and insole pressure threshold judgment. The difference between these three methods lies in the conditions that trigger the touchdown judgment; the method for updating the position after triggering the touchdown condition is consistent. ${\vec{V}}_{change}$ is the 3D vector from the touchdown foot pointing (${\vec{P}}_{t}$) to the suspended foot (${\vec{P}}_{s}$). We defined two bool variables, $TD_{R}$ and $TD_{L}$, representing the touchdown status of the right or left foot, respectively. Assuming the right foot touches the ground, the action vector is given by Equation (1); the action vector for the left foot touchdown is given by Equation (2). Finally, the location of the current suspended foot is given by Equation (3):

(1) $$\begin{array}{c} {\vec{V}}_{change}={\vec{V}}_{R2L}={\vec{P}}_{L}-{\vec{P}}_{R},\;if\;TD_{R}=1, \end{array}$$

(2) $$\begin{array}{c} {\vec{V}}_{change}={\vec{V}}_{L2R}={\vec{P}}_{R}-{\vec{P}}_{L},\;if\;TD_{L}=1, \end{array}$$

(3) $$\begin{array}{c} {\vec{P}}_{s}={\vec{P}}_{t}+{\vec{V}}_{change}, \end{array}$$

where ${\vec{P}}_{R}$ and ${\vec{P}}_{L}$ are the positions of the right and left foot, respectively.

The problem is how to choose from Equations (1) and (2) to find ${\vec{V}}_{change}$. In the following sections, three touchdown judgment methods are compared.

### 2.1. Judgment by Height of Left and Right Foot—Only IMU

Day to day, the human body, especially in an indoor environment, mostly experiences flat walking situations. There is a change in the vertical drop when walking, and the lowest point of both feet is the moment the human foot touches the ground. Hence, we can judge which foot is touching the ground by comparing the vertical height drops, with the following Equation (4):

(4) $$\begin{array}{c} TD_{R}=1,\;if\;{\vec{P}}_{Lz}>{\vec{P}}_{Rz},\\ TD_{L}=1,\;if\;{\vec{P}}_{Rz}>{\vec{P}}_{Lz}, \end{array}$$

where ${\vec{P}}_{Rz}$ and ${\vec{P}}_{Lz}$ are the z axis positions of the right and left foot, respectively.

The advantage of the both feet level height comparison algorithm is the simplicity of the judgment, the low consumption of computing resources and the ability to quickly determine which foot is in contact with the ground. However, there is a greater possibility of misjudgment, mainly reflected in the following three aspects:

1. The purely flat walking area in the daily state is too idealized to guarantee the horizontal state at the time of walking;
2. The angle error and error connection caused by the measurement error will lead to the jumping error between the actual height position of the feet and the calculated height position, which will lead to the estimation of the height of the feet not being absolutely accurate;
3. The judgment of height comparison between the feet during walking and the moment of contact between the foot and the ground do not coincide exactly. During the gait cycle, only the middle of the support phase is identifiable, as the foot in contact with the ground does not produce large slips, while other transition moments produce a situation where the position of the foot in contact with the ground moves relative to the ground, so it will lead to incorrect judgments of the time of full contact with the ground.

### 2.2. Zero-Speed Detection and Positioning of Foot Jitter Acceleration—Only IMU

**Figure 1:**
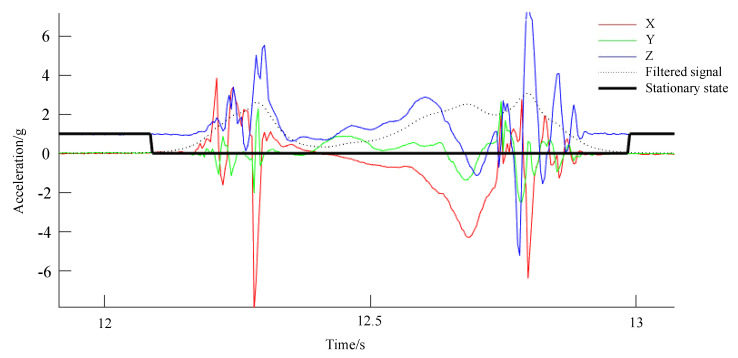
Acceleration and stationary during walking.

According to the law of the data in the gait cycle, the acceleration data of the foot will be close to the stationary state when the foot is in a state of touching the ground [30,31]. After through filtering and threshold judgment, the triaxial acceleration vectors can obtain the feet alternately time of quasi stationary state with the ground. As shown in Figure 1, when the stationary data is 1, it is judged to be the moment when the foot touches the ground and locks with the ground [32], determined by removing the 3D component of gravity in the absolute space of the sensor, as shown by Equation (5):

(5) $$\begin{array}{c} TD_{R}=1,\;if\;\left|A_{R}\right|<A_{a},\\ TD_{L}=1,\;if\;\left|A_{L}\right|<A_{a}, \end{array}$$

where $\left|A_{L}\right|$ is the 3D acceleration value of the left foot, $\left|A_{R}\right|$ is the 3D acceleration value of the right foot, $A_{a}$ is the modulus length of the threshold foot acceleration for determining the stationary landing foot and scalars $\left|A_{R}\right|$ and $\left|A_{L}\right|$ represent the right and left foot, respectively.

The advantage of the foot acceleration locking algorithm is that the state at the moment of touchdown can be judged more quickly by acceleration and has the characteristic of a more obvious data cycle. However, in the process of three-dimensional fixed measurement, if the absolute acceleration analysis is adopted, that is, containing the numerical component of gravitational acceleration in three axes, the numerical intelligence of the three-axis components is not completely consistent with the data thresholds of different fixed positions and attitudes. If the net three-dimensional acceleration method with gravitational acceleration removed is used to measure the data, it is necessary to calculate the spatial attitude of the data and eliminate the projection component of gravity, which is computationally intensive. As the touchdown foot is not completely stable, this method will experience acceleration jumps on the uneven and partially rugged ground, resulting in an incorrect moment of touchdown judgment.

### 2.3. Judgment by Net Data of Foot Pressure Insoles—IMU + Insole

Generally, the method of using pressure shoes to trigger and determine the contact point is more accurate than the IMU method. The plantar pressure value is monitored cyclically, and considered a touchdown state when it exceeds the threshold, as shown in Equation (6):

(6) $$\begin{array}{c} TD_{R}=1,\;if\;MaxPressure_{R}\;>\;threshold,\\ TD_{L}=1,\;if\;MaxPressure_{L}\;>\;threshold, \end{array}$$

**Figure 2:**
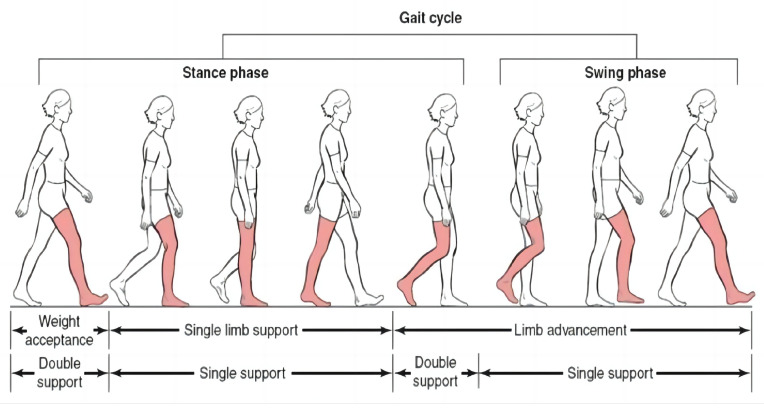
The phases of a gait cycle.

Figure 2 shows the phases of the gait cycle starting with the right leg as an example, which was mainly divided into four support types [33]. In the double support phase, where both feet are touching the ground, the foot may slide on the ground. Then, in the single support phase, the right foot touches the ground with no movement. In the third phase (another double support phase), the left foot begins to touch the ground. In the last phase, the left foot becomes the single support foot.

Due to the uniformity of the foot morphology, it is common to use the judgment method of plantar pressure [34] net value for a quick moment-to-moment judgment of whether there is a touchdown. However, this will cause a large error in the judgment when the soles of the feet and the ground slide or the ground is relatively rugged and uneven.

## 3. Materials and Methods

### 3.1. System Architecture

**Figure 3:**
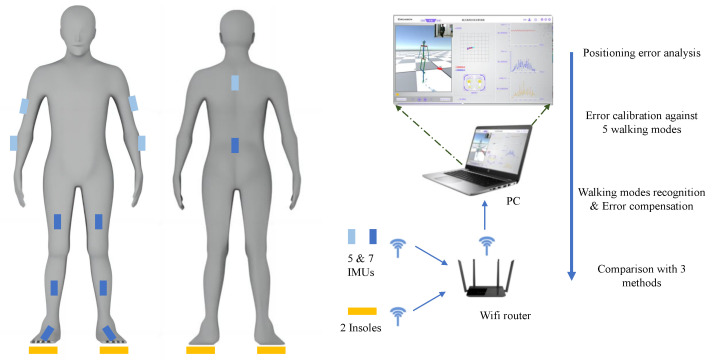
The architecture of the WSN system and the framework of this paper.

Twelve IMUs and a pair of insoles with pressure sensors were combined to collect data, as shown in Figure 3. However, in fact, we utilized seven IMUs marked with dark blue to calculated the $P_{R}$ and $P_{L}$, and insoles marked with orange to compensate for theerror. Each cross symbol represents the location of the IMU, and every pressure insole is placed inside corresponding shoe. All data needed are transmitted to the PC through WiFi with the UDP protocol, and then analyzed in the software. The data transfer rate is 50 Hz. By analyzing the UDP protocol data of wireless sensor, data delay rate is less than 10 ms. All data are synchronized by timestamp alignment in multiple sensor data frames in the motion capture software, and the error of the synchronized acquisition trigger time is controlled within 20 ms. The synchronized data are processed to form a 3D structure of the human rod connection and a plantar pressure distribution image of both feet in self-developed gait analysis software. This complete WSN system [35] can collect and analyze data during human walking, identify the contact points of the foot and compare the actual walking path with the reconstructed path data.

**Figure 4:**
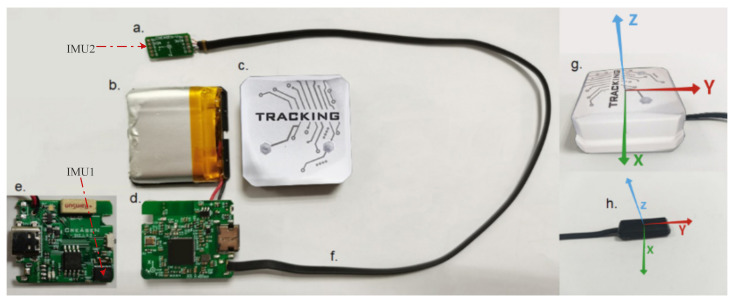
Structure of the IMU and its coordinate system.

The posture measurement system used in this work was self-designed, which is called TRACKING${}^{\mathrm{TM}}$ and typed as CRE-CC02, with 0.81° absolute angle accuracy tested by the third-party institution CHANGCHENG INSTITUTE OF METROLOGY & MEASUREMENT. It is a nine-axis IMU with three axes, i.e., accelerometer (range: ±16 g), gyroscope (range: $\pm 2000^{\circ }$/s) and magnetometer, available for multi-dimensional data fusion and posture recognition relative to the ground coordinate. As shown in Figure 4, IMU can be connected to the main chip (an MCU supporting wireless communication at the center of Figure 4d) by the IIC bus in two ways. One way is to integrate the chip onto the board, as shown in Figure 4e. Another way is to connect the module to the board through wires (see Figure 4a,d,f). There are four combinations available depending on the way and location, and we can choose from them according to actual needs. The product is powered by a lithium-ion rechargeable battery (see Figure 4b). The appearance packaged in a shell (see Figure 4c) and coordinate system definition is shown in Figure 4g,h. IMU’s data, including quaternions, three axes of acceleration and three axes of angular velocity, are sent to the PC at each sampling period. The size of the module in Figure 4g is 37 mm × 37 mm × 14 mm, while the size of the module in Figure 4h is 25 mm × 10 mm × 4.5 mm.

**Figure 5:**
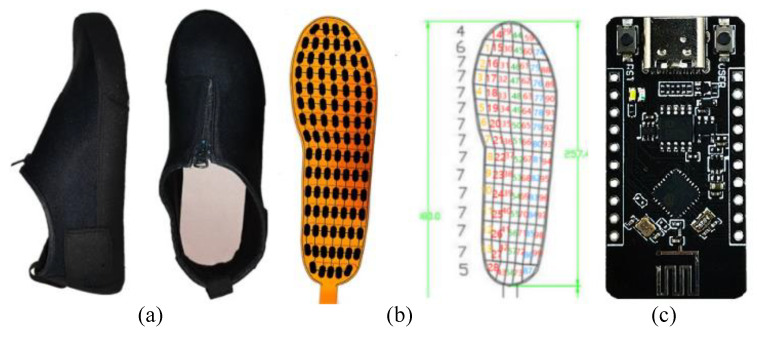
Structure of shoes and insoles for experiments. (**a**) Shoes; (**b**) Pressure insole; (**c**) Arduino MCU.

**Figure 6:**
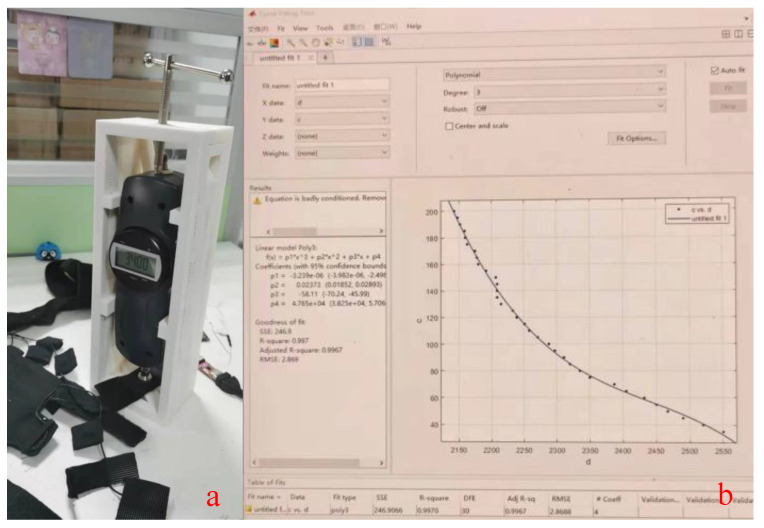
Insoles’ pressure numerical calibration. (**a**) Pressure calibration; (**b**) Fitted pressure curve.

While traditional plantar pressure insoles use eight or fewer pressure sensors, we chose a high-resolution pressure insole (RPPS-99, LEGACT Technology Co., Ltd, Shenzhen, China) with 99 points (measuring range: 0.1–10 kg, durability: 1 million times) for plantar-pressure-data acquisition and analysis, as shown in Figure 5. Each insole is equipped with an ESP32 Arduino to collect the AD value and send data to a PC by using WiFi. The figure also shows the distribution of plantar-pressure sensors and shoes used for experiments. Due to the fact that insoles are not self-designed, the first step before the experiment is to calibrate insoles using a pressure gauge (WDF-500, Wei Du Electronics Co., Ltd, Wenzhou, China) with a measurement of 500 N and accuracy of ±1%. As shown in Figure 6a, the pressure gauge is pressurized to the insole’s sense point by rotating the screw at the top end. Then, 14 bit of AD voltage of the single point sampled by 50 Hz is printed in real time through a serial port. The collected measurement values corresponding to pressure values of 0–200 is calibrated using the MATLAB curve fitting tool, as shown in Figure 6b. The result shows that the mathematical relationship between the pressure value and the AD value of the insole is a cubic polynomial. After completing the calibration, multiple plantar pressure data can be collected. In our experiments, the point at which the pressure is greater than the threshold (5N) will have its value recorded, with an acquisition range of 5–200 N.

### 3.2. Error Analysis of the Foot Touchdown Position

#### 3.2.1. Error in ${\vec{P}}_{change}$

**Figure 7:**
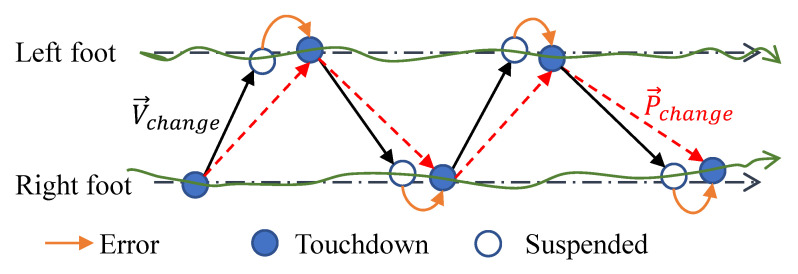
Principal error analysis of the algorithm.

Each of the above three methods (see Section 2) has its own advantages and disadvantages, but none of them have been able to solve existing principal errors. In Figure 7, it is assumed that both feet move along the desired straight line from left to right (right arrow dotted line), and in fact, that the landing point is fitted as a spline curve (solid line with arrow), with a solid circle representing the landing point (${\vec{P}}_{t}$) and a hollow circle representing the position of one foot in the air ${\vec{P}}_{s}$ when another triggers a touchdown. The vector ${\vec{V}}_{change}$ is the change from a foot during touchdown and suspension (see Equation (3)). In theory, the real action vector connects two adjacent landing points (i.e., ${\vec{P}}_{change}$), while ${\vec{V}}_{change}$ leads to errors in analyzing the touchdown to suspended foot position.

#### 3.2.2. Error in ${\vec{P}}_{R},\;{\vec{P}}_{L}$

**Figure 8:**
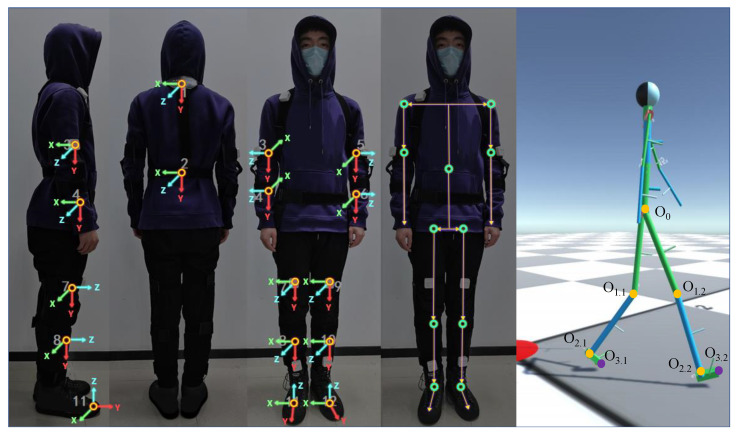
Link-frame assignment of the whole body. The first three pictures axes of IMUs at their positions, while the last two pictures are the simplified joint coordinate system.

For real-time path tracking in gait analysis, we expected to capture more accurate landing points. A three-dimensional real-time pose model of human bones [36] is constructed in gait analysis using spatial pose equations. Here, the additional five IMU modules were not considered. Each position and posture information of lower limbs can obtained with link-frame assignment [37], as shown in Figure 8, relying on data of seven IMUs (one at the waist and six at the lower limbs, see Figure 3). Here, O${}_{0}$ represents hip joint, O${}_{1.x}$ and O${}_{2.x}$ represent the centers of articulation (knee and ankle joints) and O${}_{3.x}$ represent the end of feet. Among them, two purple marker points (O${}_{3.x}$) represent the foot position (i.e., ${\vec{P}}_{R},\;{\vec{P}}_{L}$), which is from the transformation matrix, as shown in Equation (7) within the motion capture system:

(7) $${}_{3}^{0}R={\;}_{1}^{0}\;R_{2}^{1}R_{3}^{2}R,$$

where ${\vec{P}}_{R}$/${\vec{P}}_{L}$ is from the last column of a matrix ${}_{3}^{0}R$, which represents O${}_{3.x}$ relative to O${}_{0}$, and *x* refers to both legs.

**Figure 9:**
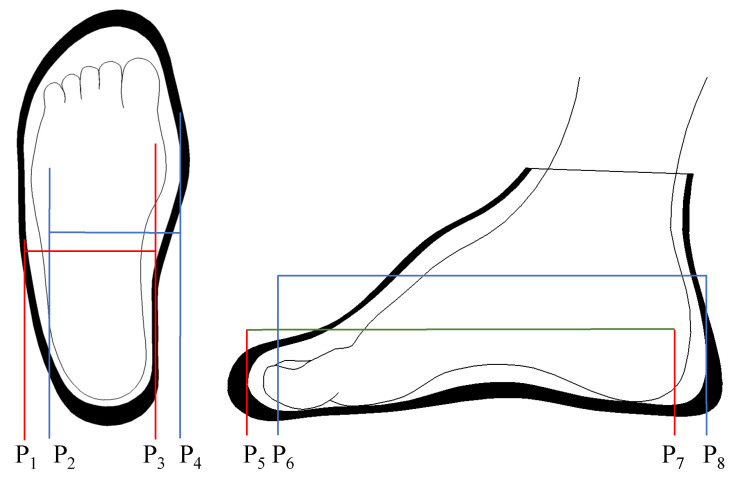
Possible relative positions of a foot in a shoe during walking.

Since the wrapping between the shoe and the foot is a loosely fixed relationship, the position of the foot relative to the shoe is not fixed during walking. As shown in Figure 9, $P_{1}$ to $P_{3}$ is the leftmost position of the foot in the shoe, $P_{2}$ to $P_{4}$ is the rightmost position, $P_{5}$ to $P_{7}$ is the foremost position and $P_{6}$ to $P_{8}$ is the rearmost position. The movement of the feet relative to the calves (in Figure 8, O${}_{3.x}$ is relative to O${}_{2.x}$, and *x* refers to both legs) only involves rotation, without a translation motion; the IMU fixed on the shoe has a displacement relative to the foot during walking, which leads to errors in calculating ${\vec{P}}_{R},{\vec{P}}_{L}$ by a kinematic transformation matrix.

### 3.3. Proposed Improvement—IMU + Insole

In order to improve the accuracy of real-time path tracing in gait analysis, considering errors in ${\vec{P}}_{R},\;{\vec{P}}_{L}$ and ${\vec{P}}_{change}$, this paper improves the threshold judgment method of insole pressure (see Section 2.3).

**Figure 10:**
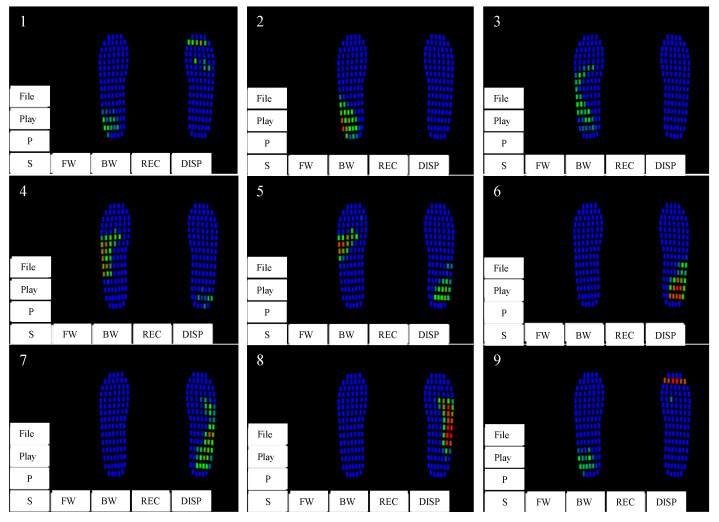
Plantar pressure (2 × 99 points) analysis during walking. Steps 1–9 represent the plantar pressure distribution during a gait cycle. Button abbreviation: P(Pause), S(Stop), FW(Forward), BW(Backward), REC(Record), DISP(Display).

By using pressure insoles with high spatial resolution, we can more accurately locate the force point of the foot, as shown in Figure 10. Obviously, there are different force points when we walk on different paths, such as going straight, turning, etc. The walking mode can be realized through the position relationship of the maximum pressure point on the front and back soles of both feet during walking.

**Figure 11:**
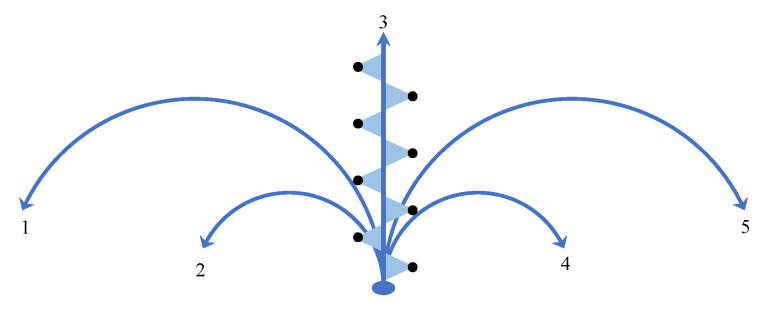
Five walking modes for error compensation. 1. Left turn with r = 4 m; 2. Left turn with r = 2 m; 3. Go straight; 4. Right turn with r = 2 m; 5. Right turn with r = 4 m. The black dots represent the straight line gait.

**Figure 12:**
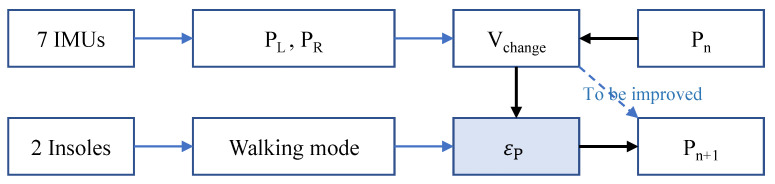
Flow of the touchdown position calculation with compensation.

After analyzing the plantar-pressure points in the process of walking, we carried out more fine-error-position compensation. We have defined five walking modes: straight forward walking, small angle left turn, small angle right turn, large angle left turn and large angle right turn, as shown in Figure 11. The small turning radius is 2 m, and the large turning radius is 4 m. In the path tracking process, not only should the touchdown trigger be identified, but also the walking mode. According to the real-time recognized walking mode, we compensate for the error of each step to achieve higher positioning accuracy, as shown in Equation (8). The flow of the real-time compensation algorithm realized with the software is shown in Figure 12.

(8) $${\vec{P}}_{s}={\vec{P}}_{t}+{\vec{V}}_{change}+\varepsilon_{P},$$

where $\varepsilon_{P}$ is the compensation value, which depends on the recognized walking mode.

The rules table for walking modes recognition and compensation values corresponding to the five modes were obtained with the experiments. All three participants had normal arched feet. Each person walked 50 steps at a time and repeated this three times along each path, for a total of 45 trials. We firstly marked a series of points on the path, with a jagged distribution of an average of 0.5–0.6 m intervals, as shown with the black points in Figure 11. Other paths are similar. Participants were asked to step on the marker point for each foot landing. By comparing the measured values ${\vec{P}}_{L},{\vec{P}}_{R}$ with the actual value of the corresponding marked point, we were able to obtain the error compensation values for both feet in each walking mode. Finally, we took the average error of nine trials as the compensation values for each mode, as shown in Table 1.

**Figure 13:**
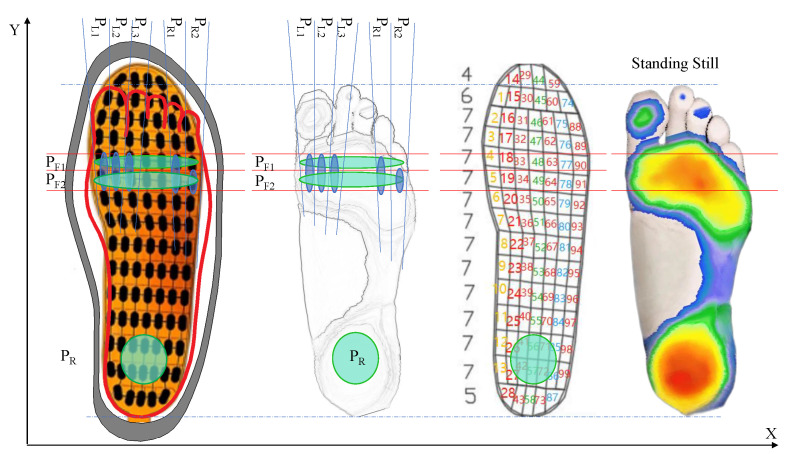
Coordinate position of the pressure insoles and possible position of max force point under when standing still (Red: high pressure; Blue: low pressure; white: no pressure). The number 1–99 represent the position of each sensor in a insole.

Furthermore, we established the coordinate position of the high-resolution pressure insole, as shown in Figure 13. By comparing the foot pressure distribution characteristics under five walking modes, areas with obvious recognition were identified. $P_{F1}$ and $P_{F2}$ positions were selected latitudinally, and $P_{L1}$, $P_{L2}$, $P_{L3}$, $P_{R1}$ and $P_{R2}$ longitudinally. The fourteen pressure sensing points covered are the key data for determining the walking mode. The discriminant criteria are summarized in Table 2 after the experiments. BW is the body weight of the human body. When the Step detected is less than the threshold $L_{w}$ (15 cm here, because it can filter out the situation of walk dragging along the ground), and the total pressure at the relevant positions exceeds 0.7 times the body weight, it can be considered to be in a stationary standing state. FL represents the left foot pressure, FR represents the right foot pressure and the subscripts correspond to the pressure area in Figure 13. For example, $FL_{PR1}>th$ means that when the pressure value in the PR1 area of the left foot is greater than the threshold, it is determined that the left foot is in a 4 m radius right turn mode. Based on Table 1, the compensation value [x, y] is equal to [−0.94, −0.52]. $\sum FL_{PRi}$ is the sum of $FL_{PR1}$ and $FL_{PR2}$. `&’ means logical `and’; `|’ means logical `or’. th is the pressure threshold for triggering judgment.

### 3.4. Comparison Experiments

**Figure 14:**
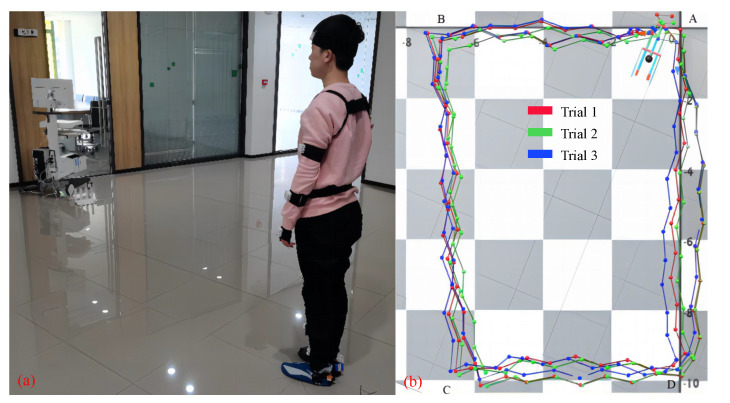
Data collection and path tracking in the walking experiments. (**a**) Walking experiments; (**b**) Real-time positioning and track record.

To verify that the improved compensation algorithm is more accurate than the previous three methods (see Section 2), we designed repeated experiments of walking on a flat ground. In the experiment, we asked each subject to wear wireless IMUs and wireless plantar pressure insoles and walk counterclockwise from point A along a straight line to points B, C and D, and finally back to point A. Figure 14a shows a top view of the experimental site, where the checkerboard grid represents the dimensions of the floor tiles, each measuring 400 mm × 400 mm, with AB and CD measuring 7 × 400 mm and BC and DA measuring 10 × 400 mm. We collected the motion capture and plantar pressure synchronization data from four healthy representative subjects (see Table 3) of different body shapes, 10 times/person, for a total 40 trials.

During each experiment, the data of all IMUs and insoles are synchronously collected by the computer, and the real-time virtual motion capture of IMU can be seen with the gait analysis software, as shown in Figure 14b. After all the experiments were completed, we used four methods to reconstruct the walking path (see right side in Figure 14). Finally, we compared the deviation between the walking paths fitted by the four methods and the preset test rectangular path.

## 4. Results

For the convenience of description, we have agreed on the identification of four methods as follows:

Method I: Judgment by height of both feet;
Method II: Zero-speed detection;
Method III: Net data of plantar pressure;
Method IV: Improved compensation algorithm.

**Figure 15:**
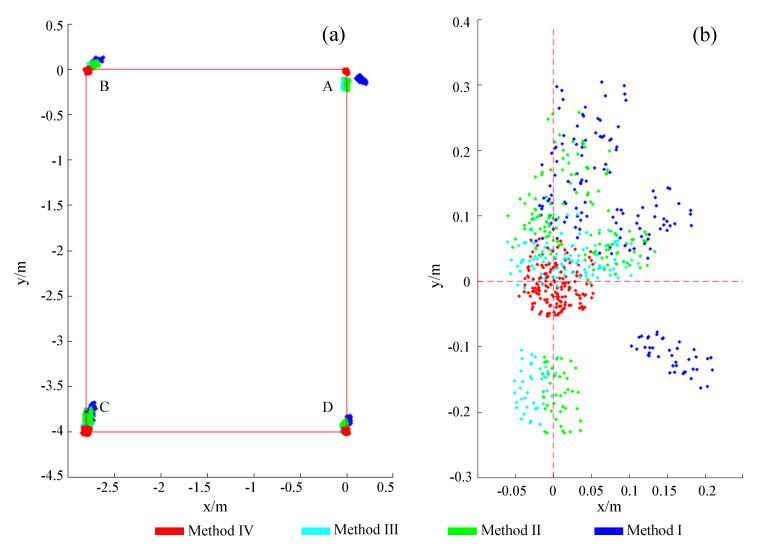
(**a**) Absolute positions of the four methods; (**b**) The net deviations of all points.

**Figure 16:**
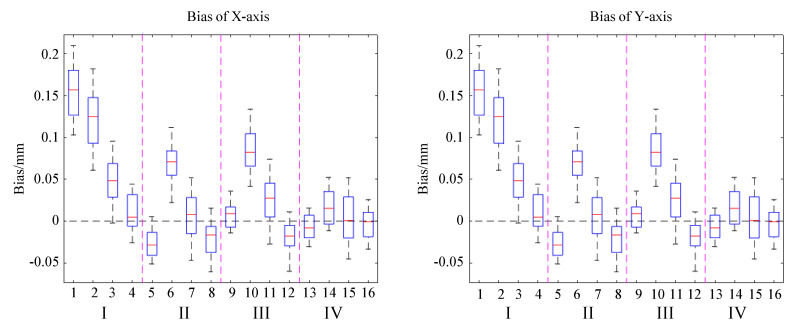
Comparison of the position bias by four methods in the X, Y direction.

After using these four methods to process data and reconstruct paths separately, we obtained position values of the four marked points A, B, C and D. It should be noted that point A data represent the value when walking back to the starting point. In Figure 15, where blue, green, cyan and red correspond to the data points results of the above four methods, Figure 15a shows the absolute positions at each point, and Figure 15b shows the net bias of all points after subtracting the absolute positional deviation. Method IV outperforms the other methods in terms of absolute value response and net deviation in both mean position and dispersion. Figure 16 shows the mean and net deviation results in the x-direction and y-direction at points A, B, C and D (four boxes from left to right in each subblock) with the four methods. Both figures show that the fourth set of data outperforms the previous sets in terms of mean, median and deviation values.

By comparing the mean, median standard deviation and variance of the four points under different methods, as shown in Table 4, we found that Method IV has the lowest mean/median bias and variance at each point. This result indicates that the values obtained by the fourth method are closer to the target point than the previous three methods.

The original data of A, B, C and D obtained by the four methods were statistically analyzed, and the data of central tendency and dispersion degree of each group of data were obtained, as shown in Table 4. Comparing the mean and median values obtained by the four methods at points A, B, C and D, we can see that the mean and median values obtained by the second method are smaller than those obtained by the first method, the mean and median values obtained by the third method are smaller than those obtained by the second method and the mean and median values of X and Y obtained by the fourth method are closer to 0 compared to those obtained by the first three methods. This indicates that the values obtained by the fourth method are closer to the target point than the first three methods.

## 5. Discussion and Conclusions

By comparing the standard deviation and variance values obtained by the four methods at four points A, B, C and D, it can be found that the standard deviation and variance values of X and Y obtained by the second method are less than those of the first method, the values obtained by the third method is less than that of the second method and the values obtained by the fourth method are the least. The results show that the fourth method has a lower degree of data dispersion, shown by the statistical results in the scatter diagram, see Figure 15.

In our research, we proposed a improved feet positioning compensation method, using a practical multi-sensor data acquisition and analysis system. By sensing the walking mode through high-spatial-resolution plantar pressure, the system automatically matches the compensation values obtained from the experiments. Compared to other methods, it has higher accuracy to recognize touchdown and trace path in gait analysis. In a sense, this is a compensation method based on statistical error [38], so it can achieve even better results under test conditions within the preset mode range. It was reported that none of 17 proposed algorithms can be generally preferred over the others in gait event detection from IMU measurements, taking the IMU position, target variable and computational approach into account [39]. Our method, error compensation against waking modes, is generally applicable to those algorithms for improving positioning accuracy.

This paper only tested the four methods under situation of walking on a flat ground. As a next step, we will also carry out experiments on human–object hybrid recognition [40] for indoor staircase scenes, corridor scenes, slope scenes, door scenes, etc. When the amount of data for various walking modes is large enough, we will use machine learning and deep learning algorithms to achieve end-to-end automated walking mode recognition and position compensation systems.

When using IMU and pressure insoles for tracking and positioning, there are several limitations that affect the accuracy limit:

1. Fixed bias and motion shift of sensors: the solution to this is to fix the module tightly to the skeletal position of the body limbs and avoid fixing it to unstable muscle parts.
2. Interference of magnetic field disturbance to IMU: it is suggested to avoid using this method in areas with strong magnetic fields and to keep it away from magnetic fields in the process of preservation.
3. Partial bending of insoles: the solution to this is to use the fourth method to support the process of phase.
4. Sliding of insoles in shoes; tying the shoelace tightly may be better.

The first two factors affect all four methods, while the latter two factors are only related to Method III and IV. That is to say, the method we propose is related to all four factors. These errors can be minimized as much as possible, though they cannot be completely eliminated, by improving the experimental design, equipment, environment, etc.

The high-precision wearable positioning method can break through the limitations of indoor positioning that only relies on the single technology of RF, can be used in a scene without an RF layout and with a low RF signal and does not require higher positioning accuracy. The method in this paper improves the real-time positioning accuracy of wearable systems, which has a positive significance for related fields, such as gait analysis, wearable device applications, etc. Compared with the external positioning reference systems, wearable active positioning solution can rely on its own actual position and dynamic real-time offset to achieve real-time positioning. It will provide positioning support to rescue operations, in scenarios where external infrastructure is damaged, due to fires, earthquakes, etc.

In this paper, a positioning method based on IMU and pressure insole for path tracing was improved. We collected real-time data of whole-body movement and plantar pressure distribution. After repeated experiments and statistical analysis, the results show that this method has a higher accuracy than the previous three methods. Our system, implemented based on the improved method, can achieve real-time (within 20 ms) and high-precision (within a 0.05 mm standard deviation) indoor positioning and path tracing. This study has certain practical significance.

## Figures and Tables

**Table 1:** Compensation values.

$\varepsilon_{P}$/mm	Left Foot						Right Foot					
Direction	Left Turn		Forward		Right Turn		Left Turn		Forward		Right Turn	
Axis	x	y	x	y	x	y	x	y	x	y	x	y
Going straight	–		0.34	0.55	–		–		−0.23	0.62	–	
Turning r = 4 m	0.19	0.27	–		−0.94	−0.52	0.97	−0.48	–		−0.16	0.24
Turning r = 2 m	0.35	0.97			−1.28	−1.73	1.25	−1.8			−0.34	1.02

**Table 2:** Walking modes’ discriminant criteria.

Trigger Condition		Standing Still	Going Straight	Turing r = 4 m	Turing r = 2 m
Left Foot	Turn Left	–		$FL_{PR1}>th$	$FL_{PR2}>th$
	Turn Right			$(FL_{PL2}\;|\;FL_{PL3})>th$	$FL_{PL1}>th$
Both	Forward	$Step<L_{w}$ (15 cm) & ($\Sigma FL_{PRi}$ + $\Sigma FR_{PRi}$) >0.7∗BW	$Step>L_{w}$ (15 cm) & (No others trigger) & $(FL_{PF1}+FR_{PF1}-$ $FL_{PF2}-FR_{PF2})>th$	–	
Right Foot	Turn Left	–		$(FR_{PL2}\;|\;FR_{PL3})>th$	$FR_{PL1}>th$
	Turn Right			$FR_{PR1}>th$	$FR_{PR2}>th$

**Table 3:** The information of the subjects.

	Gender	Height/cm	Weight/kg	Shoe Size/mm
1	female	172	63	245
2	male	168	52.5	255
3	female	160	50	235
4	male	183	81	270

**Table 4:** Mean, median, standard deviation and variance of the four methods at the four points.

Point No. & Method No.		Mean Value		Median		Standard Deviation		Variance	
		X/mm	Y/mm	X/mm	Y/mm	X/mm	Y/mm	X/mm${}^{2}$	Y/mm${}^{2}$
A	I	0.1556	−0.1141	0.1569	−0.1134	0.0298	0.0225	0.0009	0.0005
	II	−0.0267	−0.1606	−0.0286	−0.1579	0.0154	0.0322	0.0002	0.001
	III	0.0073	−0.1733	0.009	−0.1717	0.0149	0.0373	0.0002	0.0014
	IV	−0.0063	−0.0289	−0.008	−0.0289	0.0152	0.0162	0.0002	0.0003
B	I	0.1226	0.0865	0.1247	0.0872	0.0317	0.0283	0.001	0.0008
	II	0.068	0.034	0.0709	0.029	0.0234	0.0212	0.0005	0.0004
	III	0.0844	0.0461	0.0824	0.0452	0.0255	0.0202	0.0007	0.0004
	IV	0.017	−0.0208	0.0152	−0.0204	0.0202	0.0198	0.0004	0.0004
C	I	0.0478	0.2299	0.0483	0.2207	0.0287	0.039	0.0008	0.0015
	II	0.0071	0.043	0.0077	0.0379	0.0259	0.0333	0.0007	0.0011
	III	0.0259	0.1708	0.0273	0.1704	0.0286	0.0415	0.0008	0.0017
	IV	0.0029	0.015	0.0007	0.0217	0.0283	0.0309	0.0008	0.001
D	I	0.0103	0.1145	0.0048	0.1132	0.0216	0.031	0.0005	0.001
	II	−0.0197	0.0274	−0.0166	0.0286	0.0203	0.019	0.0004	0.0004
	III	−0.0181	0.0846	−0.0178	0.0825	0.0177	0.0247	0.0003	0.0006
	IV	−0.0044	0.01	−0.0007	0.0075	0.0176	0.0178	0.0003	0.0003

## Data Availability

The data that support the findings of this study are available from the first author, [1610073@stu.neu.edu.cn], upon reasonable request.

## Abbreviations

The following abbreviations are used in this manuscript:

MEMS	Micro-Electro-Mechanical System
ZUPT	Zero-Velocity Update
MCU	Microcontroller Unit
IMU	Inertial Measuring Unit
UDP	User Datagram Protocol
WSN	Wireless sensor networks
3D	Three-dimension
PC	Personal Computer
AD	Analog to digital
